# First rib resection and corrective clavicle osteotomy using the infraclavicular approach for thoracic outlet syndrome due to clavicle malunion: A case report

**DOI:** 10.1186/s12891-025-08855-x

**Published:** 2025-07-04

**Authors:** Ryogo Furuhata, Atsushi Tanji, Taku Suzuki, Noboru Matsumura

**Affiliations:** 1 Department of Orthopaedic Surgery, Ashikaga Red Cross Hospital, 284-1 Yobe-cho, Ashikaga-shi, 326-0843 Tochigi Japan; 2 Department of Orthopaedic Surgery, Keio University School of Medicine, Shinjuku-ku, Tokyo, Japan

**Keywords:** Thoracic outlet syndrome, Clavicle malunion, First rib resection, Corrective clavicle osteotomy, Bone graft, Infraclavicular approach

## Abstract

**Background:**

Thoracic outlet syndrome can develop following the malunion of a clavicle midshaft fracture. To date, thoracic outlet syndrome complicated by clavicle malunion is typically treated with either first rib resection or corrective clavicle osteotomy; however, there have been no reports of these two procedures being performed simultaneously using the same approach. We present the first documented case of thoracic outlet syndrome caused by clavicle malunion treated by simultaneous first rib resection and corrective clavicle osteotomy through a single infraclavicular approach.

**Case presentation:**

A 46-year-old woman presented with numbness and muscle weakness in the left upper limb, which worsened with 90º abduction external rotation of the shoulder joint. She had a history of conservative treatment for a left clavicle midshaft fracture 21 years earlier. Magnetic resonance imaging taken with upper extremity elevation revealed stenosis of the left subclavian artery at the costoclavicular space. Three-dimensional clavicle symmetry plane demonstrated that the distal fragment of the left clavicle displaced inferiorly and malunited, and left scapular depressed and retracted. The distance between the left clavicle and the first rib was up to 7 mm shorter than that on the right side. She was diagnosed with left arterial thoracic outlet syndrome caused by clavicle malunion. Using an infraclavicular approach, we performed the first rib resection and clavicle osteotomy. We inserted the first rib bone graft into the osteotomy site and performed the plate fixation. Her symptoms had resolved by two years postoperatively.

**Conclusions:**

The present case provides new information on the surgical procedure of thoracic outlet syndrome due to clavicle malunion. In our patient, the inferior displacement of malunited clavicle and the associated scapular malposition may cause narrowing of the costoclavicular space, resulting in the development of thoracic outlet syndrome. The present case demonstrates that the infraclavicular approach enables the simultaneous first rib resection and corrective clavicle osteotomy and provides reliable decompression of the costoclavicular space.

**Clinical trial number:**

Not applicable.

## Background

Thoracic outlet syndrome (TOS) is a late complication of clavicle malunion, with a frequency of up to 19% in all united clavicle fractures [1]. Conservative treatment for TOS due to clavicle malunion is often ineffective, and surgery is required [2, 3]. To date, most studies have used first rib resection [4, 5] or corrective clavicle osteotomy [3, 6–8] for surgical treatment of TOS. Although satisfactory postoperative outcomes have been reported with these techniques [5, 7], no direct comparison has been made, and the superiority of these procedures remains unclear. In addition, there have been reports of residual or recurrent symptoms after surgery [4, 6, 8]. Simultaneous performance of first rib resection and clavicle osteotomy is advocated to achieve comprehensive decompression of the costoclavicular space and minimize the risk of symptom recurrence; however, no reports are available on performing these procedures simultaneously.

We present the first documented case of TOS caused by clavicle malunion treated by simultaneous first rib resection and corrective clavicle osteotomy through a single infraclavicular approach.

### Case presentation

A 46-year-old woman presented to our hospital with numbness and muscle weakness in the left upper limb. Her symptoms had progressed gradually over 6 months. She had undergone conservative treatment for a left clavicular midshaft fracture 21 years earlier. She was a police officer and practiced classical ballet as a sport. Her symptoms were aggravated by shoulder joint abduction and external rotation. The Wright test result was positive (weakness of the radial artery pulse). Tenderness or radiation-induced pain was not observed in the supraclavicular fossa. Her grip strength (right/left) was 31/21 kg. The Disabilities of the Arm, Shoulder, and Hand (DASH) questionnaire score was 46.

**Fig. 1:**
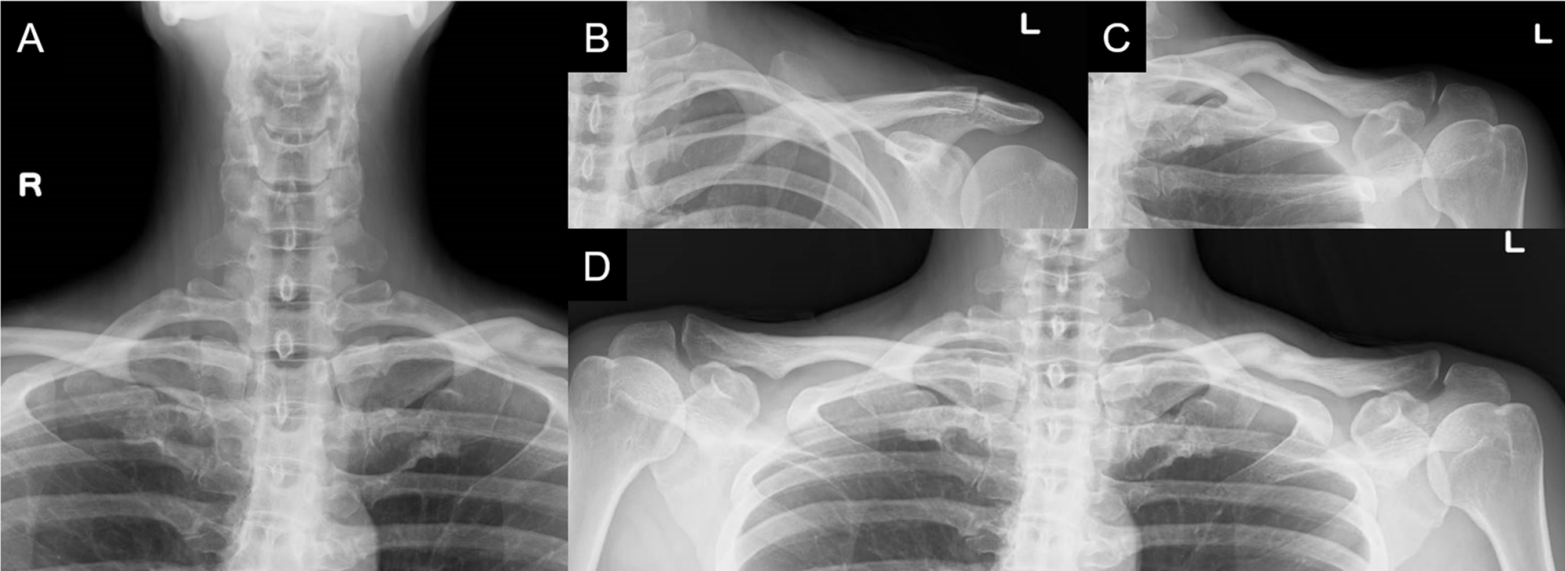
A radiograph of the cervical spine showing no cervical ribs or abnormal rib deformities (**A**). Radiographs of the clavicle showing malunion of the left clavicular midshaft fracture (**B**, **C**, and **D**)

**Fig. 2:**
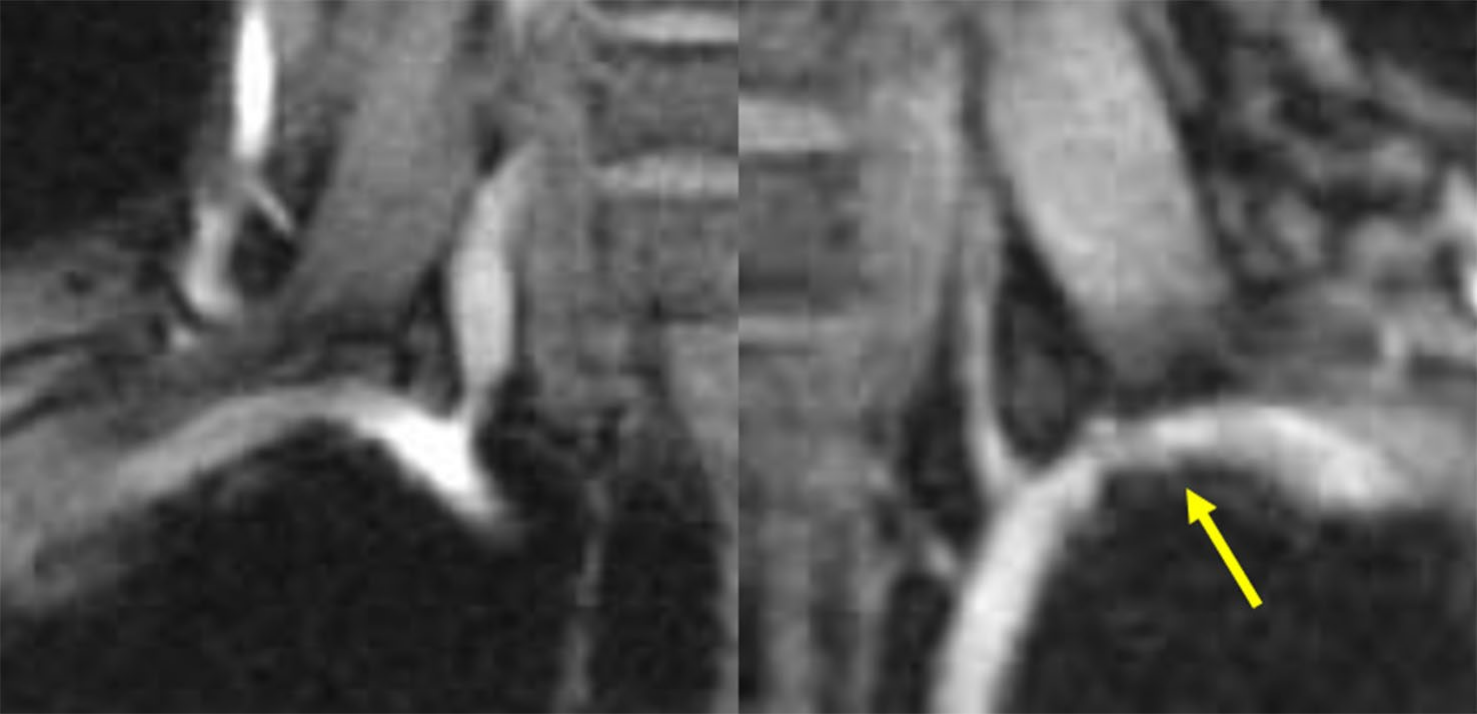
Magnetic resonance angiogram with the upper limbs in an elevated position showing a stenosis of the left subclavian artery at the costoclavicular space (arrow)

**Fig. 3:**
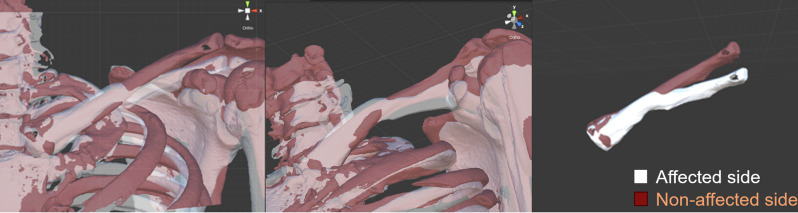
Mirroring and matching of the clavicle. We superimposed the point clouds of the three-dimensional computed tomography image of the left clavicle (white) on those of the mirrored right clavicle image using an iterative closest point algorithm (red) with respect to the first rib. The distal fragment of the left clavicle is displaced inferiorly and is malunited with the left scapula depressed and retracted. The middle panel of the figure shows that the distance between the left clavicle and the left first rib is shorter than that on the right side (up to 7 mm), indicating osseous compression of the left costoclavicular space

An anteroposterior radiograph of the cervical spine showed no cervical rib or abnormal rib deformity (Fig. 1A); however, radiographs of the clavicle showed malunion of the left clavicular midshaft fracture (Fig. 1B and C, and 1D). Magnetic resonance imaging with upper extremity elevation revealed stenosis of the left subclavian artery in the costoclavicular space (Fig. 2). In addition, we superimposed the point clouds of the three-dimensional computed tomography (CT) image of the left clavicle and those of the mirrored right clavicle image using an iterative closest point algorithm with respect to the first rib. Consequently, the distal fragment of the left clavicle was displaced inferiorly and was malunited, and the left scapula was depressed and retracted accordingly (Fig. 3). Furthermore, the distance between the left clavicle and the left first rib was up to 7 mm shorter than that on the right side, and the total length of the left clavicle was 4 mm shorter than that of the right clavicle.

Based on these findings, we diagnosed arterial TOS due to malunion of the midshaft clavicle fracture. Two months of physiotherapy failed to improve her symptoms; therefore, surgery was scheduled. Based on the previous reports [3–8], we proposed first rib resection, corrective clavicle osteotomy, or both simultaneously to the patient. Considering the occupational risk factors for TOS, such as overhead work and heavy labor, and the marked clavicle bayonet deformity with scapular malposition, we ultimately decided to perform simultaneous first rib resection and corrective clavicle osteotomy.

**Fig. 4:**
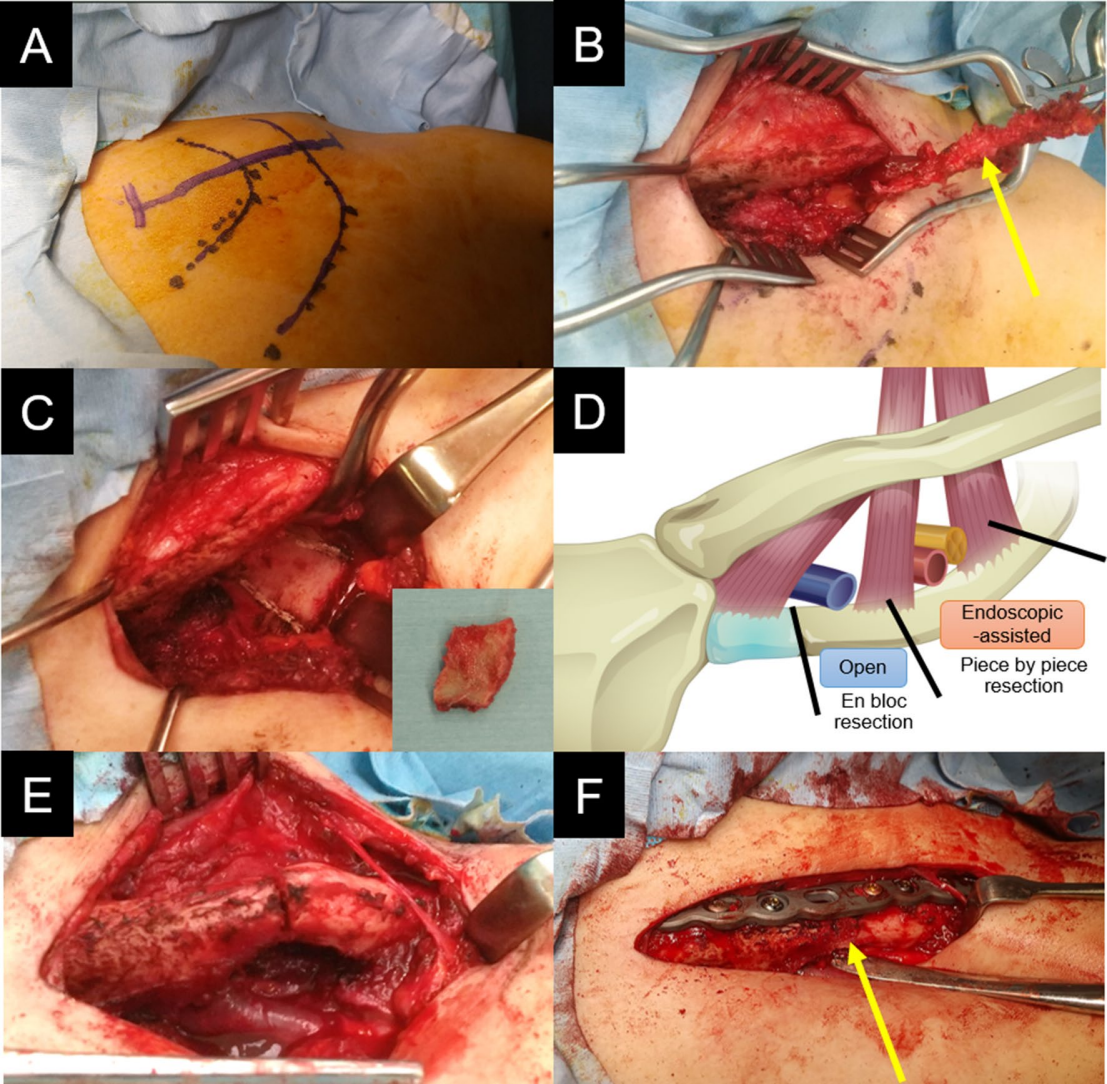
Intraoperative findings. A transverse incision was made 1 cm below the clavicle (**A**). Using the infraclavicular approach, we resected the subclavian muscle (arrow) (**B**). Under direct view, we resected the first rib in a block from the anterior margin to the origin of the anterior scalene (**C**). Under endoscopic view, we resected the first rib piece-by-piece from the origin of the anterior scalene muscle to that of the middle scalene muscle. Schematic illustration of the first rib resection (**D**). After the first rib resection, we exposed the clavicle circumferentially and performed an osteotomy (**E**). The first rib graft was fashioned into a wedge and interposed at the osteotomy site (arrow). We applied a plate on the superior surface of the clavicle (**F**)

**Fig. 5:**
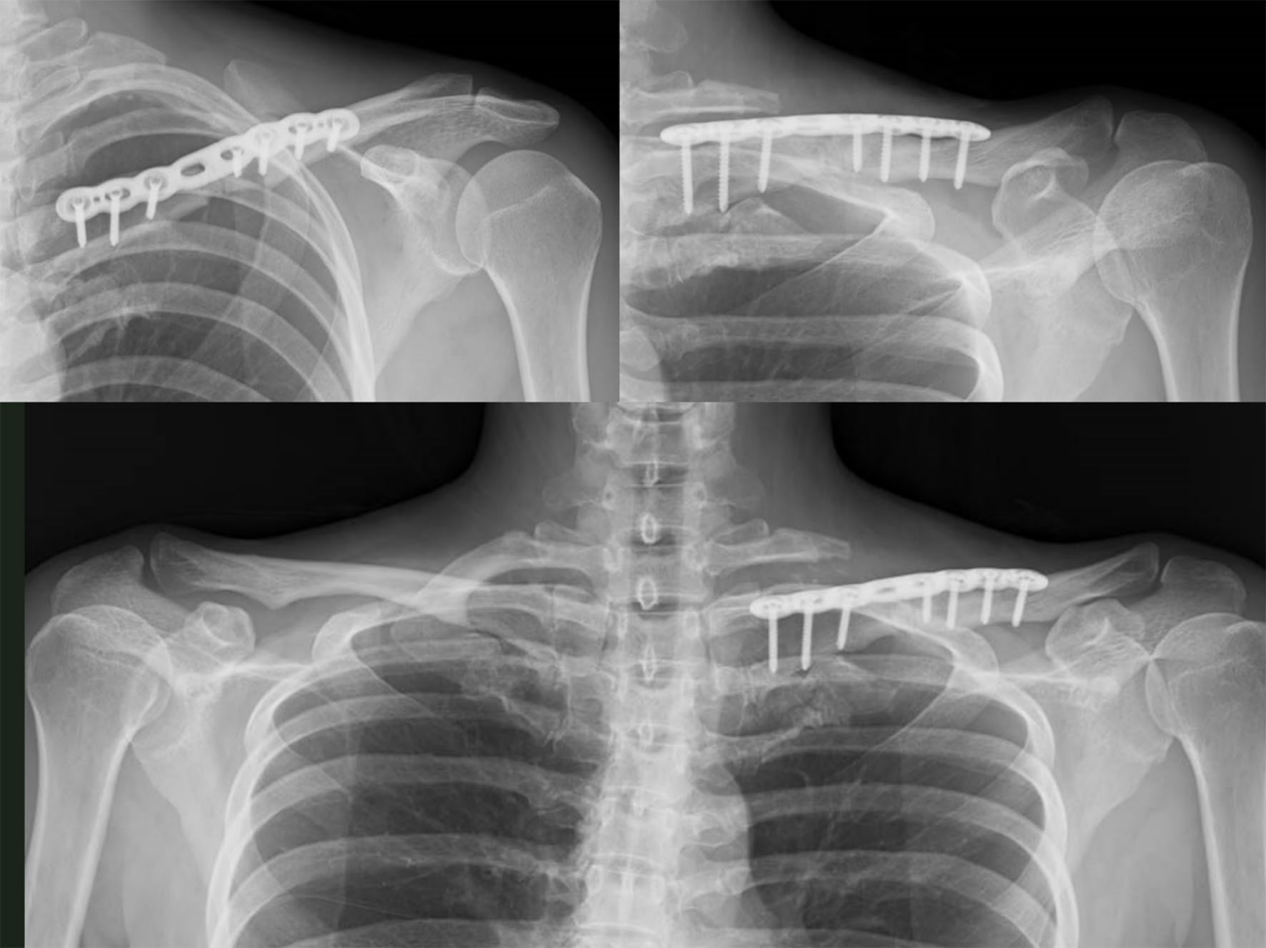
Postoperative radiographs

We identified the sternal notch, manubrium, and clavicle as bony landmarks. A 7.5 cm transverse incision was made 1 cm below the clavicle (Fig. 4A). The pectoralis major muscle was dissected from the clavicle, and the subclavian muscles were identified (Fig. 4B). Following resection of the subclavian muscle, the subclavian vein and first rib were exposed. We dissected the anterior scalene muscle from the origin of the first rib and then resected the first rib in a block from the anterior margin to the origin of the anterior scalene (Fig. 4C). A 30° oblique arthroscope was inserted through the same incision to explore the middle and posterior aspects of the first rib and middle scalene muscles. Under endoscopic view, we dissected the middle scalene muscle from the origin at the first rib and resected the first rib piece-by-piece using a Luer rongeur from the origin of the anterior scalene muscle to that of the middle scalene muscle (Fig. 4D). After the first rib resection, the skin incision was extended 1.5 cm distally. We performed osteotomy using a chisel after creating bone holes with a 1.8 mm Kirschner wire (Fig. 4E). The first rib graft was fashioned into a wedge and interposed at the osteotomy site. A Variax plate (Stryker, Kalamazoo, MI, USA) was applied to the superior surface of the clavicle (Fig. 4F). The postoperative radiographs are shown in Fig. 5.

From the day after surgery, active range of shoulder motion training and rotator cuff strength exercises within ˂ 90° of shoulder joint elevation were commenced during rehabilitation. Full range of shoulder motion training and scapular training were allowed five weeks postoperatively.

Numbness and muscle weakness improved immediately after the surgery. Radiographs taken two years after surgery confirmed bone union at the osteotomy site. The grip strength of the left hand recovered to 30 kg, and the DASH score was 5 at two years postoperatively.

### Discussion and conclusions

The present case presented two clinical issues. First, clavicle malunion and associated scapular malposition can compress the underlying neurovascular structures in the costoclavicular space, causing the delayed onset of TOS. Previous reports have suggested that bone protrusion at the inferior aspect of the clavicle, excessive callus formation, or scar restriction contribute to the development of TOS by compressing the subclavian artery or vein and the brachial plexus [1–12]. In our patient, preoperative CT revealed that the distal bone fragment was displaced inferiorly and was malunited, resulting in depression and retraction of the scapula, and narrowing of the costoclavicular space (normal range: 7.2–18 mm [13]) by up to 7 mm than the unaffected side. Since scapular depression and retraction are thought to lead to osseous compression of the costoclavicular space [14], it could have been a cause of subclavian artery stenosis in our patient. TOS due to clavicular malunion is characterized by delayed onset [3, 6], which concurs with the present case. Therefore, radiological examinations of the scapular and costoclavicular spaces should be considered when patients with clavicular malunion present with symptoms suggestive of TOS.

Second, the present case suggests that the infraclavicular approach, which enables simultaneous first rib resection and corrective clavicle osteotomy, is a surgical option for TOS due to clavicular malunion. Surgical techniques for TOS complicated by clavicular malunion include resection of the clavicle [2, 10], resection of the excessive callus or inferior bony protrusion [3, 6, 9, 11], first rib resection [4, 5], corrective osteotomy of the clavicle [3, 6–8], and scalenectomy [12]. Resection of the clavicle allows for reliable decompression of the costoclavicular space; however, it can result in postoperative instability of the shoulder girdle [7, 11]. Resection of excessive calluses is a simple and less invasive technique; however, the degree of decompression is difficult to determine and sometimes requires intraoperative finger pulse monitoring [11]. First rib resection allows scalenectomy in addition to the decompression of the costoclavicular space; however, postoperative stenosis between the malunited clavicle and second rib can occur, requiring corrective clavicle osteotomy [4]. Corrective clavicle osteotomy enables decompression of the costoclavicular space and recovery of the clavicular length, which has yielded satisfactory postoperative outcomes [7]; however, cases of postoperative occlusive thrombus recurrence [8] and residual symptoms [6] have been reported. Therefore, simultaneous first rib resection and corrective clavicle osteotomy are desirable to ensure decompression of the costoclavicular space and prevent symptom recurrence.

The infraclavicular approach is a surgical approach for first rib resection for TOS. This approach enables easy access and direct resection of the anterior component of the thoracic outlet, including the subclavian muscle, costoclavicular ligament, anterior scalene muscle, and the anterior aspect of the first rib [15–17]. However, due to the difficulty in accessing the posterior aspect of the first rib and the middle scalene muscle, the indication has been limited to venous TOS [15–19]. Recently, Suzuki et al. developed a surgical approach that allows visualization and resection of the posterior aspect of the first rib and middle scalene muscle with endoscopic assistance, thereby extending the indication to neurological TOS [20] and TOS due to first rib hypoplasia [21]. Despite this recent advance using an endoscopic technique, this approach remains contraindicated in cases requiring extensive venous reconstruction [17], those requiring neurolysis due to a narrow working space [20], and cases of TOS due to the cervical rib or the short first rib hypoplasia [21]. Our surgical procedure had two major advantages. First, simultaneous first rib resection and corrective clavicle osteotomy can be performed through the same skin incision. It is difficult to perform clavicle corrective osteotomy with a trans-axillary or posterior approach, which are other common approaches used for first rib resection. The infraclavicular approach provides excellent visualization and exposure of the subclavian vein in a shallow surgical field [16, 20, 22], enabling direct protection of the subclavian vein during clavicle osteotomy. Second, this technique enables the use of a bone graft block obtained from the first rib for corrective clavicle osteotomy. In a previous report on corrective osteotomy, the iliac crest or the inferior callus was used as the bone graft [6]; however, donor site complications are often a problem with iliac bone grafts, and clavicle length recovery may be inferior with bone grafts from the callus. Use of the first rib as a graft bone has not been reported; however, autogenous rib grafts for cervical spine surgery have shown union rates comparable to those of iliac crest grafts but with fewer donor site complications [23]. Rib grafts have circumferential cortices, and they are believed to be more resistant to bending and rotational forces than iliac crest grafts [23]. The infraclavicular approach can harvest a 2 cm block of bone graft from the first rib; therefore, this technique can also be indicated in patients with clavicle malunion or clavicle nonunion with severe shortening.

**Fig. 6:**
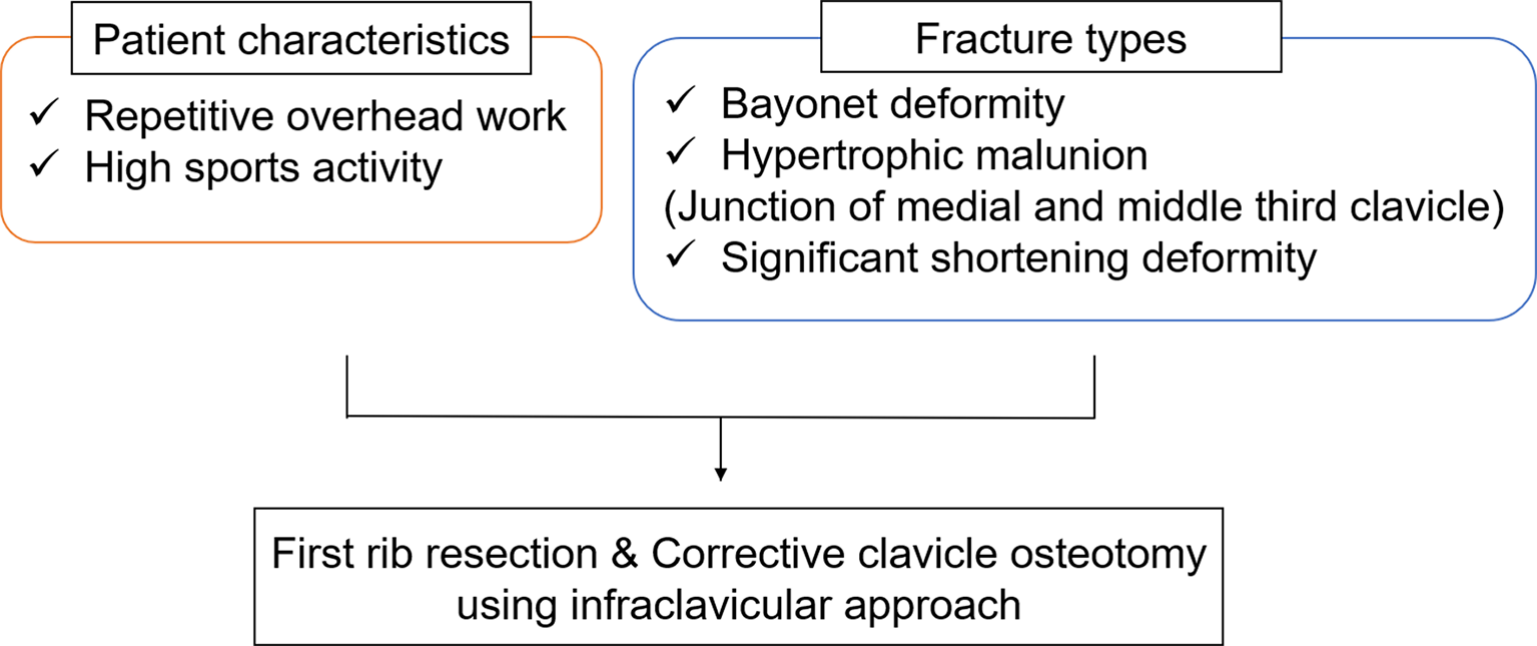
Flow diagram showing the indications for simultaneous surgery. Patients with two risk factors (patient characteristics and fracture types) may experience residual or recurrent symptoms of thoracic outlet syndrome with first rib resection or corrective clavicle osteotomy alone; therefore, simultaneous surgery is recommended

The indications for the proposed simultaneous surgery require the consideration of two factors: patient background and fracture type. A previous report showed that a patient with high overhead sports activity developed TOS due to traumatic scar tissue formation despite achieving anatomical reduction and bone union by osteosynthesis for a clavicle midshaft fracture [5]. Therefore, in patients with risk factors for TOS, not only corrective clavicle osteotomy but also first rib resection is necessary. Additionally, clavicle bayonet deformity or hypertrophic malunion at the junction of the medial and middle thirds of the clavicle can cause stenosis between the malunited clavicle and the second rib after first rib Sects. [1, 4]; therefore, corrective clavicle osteotomy is recommended in these cases. Furthermore, in cases with clavicle shortening deformity, first rib resection alone cannot restore the clavicle length to improve shoulder function [7]. Based on these reports [1, 4, 5, 7], the indications for simultaneous surgery are shown in Fig. 6. Our patient is a police officer with overhead sports activity who presented with bayonet deformity at the junction of the medial and middle thirds of the clavicle, making her a candidate for simultaneous surgery.

This case report had some limitations. First, we did not evaluate the patency of the subclavian vessels and the presence of thrombus postoperatively using duplex scanning or CT angiography. Second, this is a single-case report, which limits the generalizability of the findings. A comparative study with a larger number of cases will be needed to evaluate the usefulness of this procedure.

In summary, the present case provides new information regarding the surgical procedure for treating TOS due to clavicular malunion. In our patient, inferior displacement of the malunited clavicle and associated scapular malposition may have contributed to the narrowing of the costoclavicular space, resulting in the development of TOS. The present case demonstrates that the infraclavicular approach enables simultaneous first rib resection and corrective clavicle osteotomy, providing reliable decompression of the costoclavicular space.

## Data Availability

Data supporting the findings of this study are available from the corresponding author upon request.

## Abbreviations

CT: Computed tomography
DASH: Disabilities of the Arm, Shoulder, and Hand
TOS: Thoracic outlet syndrome
